# Secretion of *Antonospora (Paranosema) locustae* Proteins into Infected Cells Suggests an Active Role of Microsporidia in the Control of Host Programs and Metabolic Processes

**DOI:** 10.1371/journal.pone.0093585

**Published:** 2014-04-04

**Authors:** Igor V. Senderskiy, Sergey A. Timofeev, Elena V. Seliverstova, Olga A. Pavlova, Viacheslav V. Dolgikh

**Affiliations:** 1 Laboratory of Microbiological Control, All-Russian Institute for Plant Protection, St. Petersburg, Pushkin, Russia; 2 Laboratory of Renal Physiology, Sechenov Institute of Evolutionary Physiology and Biochemistry, St. Petersburg, Russia; University at Buffalo, United States of America

## Abstract

Molecular tools of the intracellular protozoan pathogens Apicomplexa and Kinetoplastida for manipulation of host cell machinery have been the focus of investigation for approximately two decades. Microsporidia, fungi-related microorganisms forming another large group of obligate intracellular parasites, are characterized by development in direct contact with host cytoplasm (the majority of species), strong minimization of cell machinery, and acquisition of unique transporters to exploit host metabolic system. All the aforementioned features are suggestive of the ability of microsporidia to modify host metabolic and regulatory pathways. Seven proteins of the microsporidium *Antonospora* (*Paranosema*) *locustae* with predicted signal peptides but without transmembrane domains were overexpressed in *Escherichia coli*. Western-blot analysis with antibodies against recombinant products showed secretion of parasite proteins from different functional categories into the infected host cell. Secretion of parasite hexokinase and α/β-hydrolase was confirmed by immunofluorescence microscopy. In addition, this method showed specific accumulation of *A. locustae* hexokinase in host nuclei. Expression of hexokinase, trehalase, and two leucine-rich repeat proteins without any exogenous signal peptide led to their secretion in the yeast *Pichia pastoris*. In contrast, α/β-hydrolase was not found in the culture medium, though a significant amount of this enzyme accumulated in the yeast membrane fraction. These results suggest that microsporidia possess a broad set of enzymes and regulatory proteins secreted into infected cells to control host metabolic processes and molecular programs.

## Introduction

Close contact between the parasite and an infected cell implies the ability of the pathogen to modify host molecular programs and biochemical processes. First, intracellular pathogens must successfully withstand protective host responses, such as induction of apoptosis [1], production of reactive oxygen species [2], autophagy, the actions of interferon-inducible GTPases [3], and the lysosomal system. Second, the parasites, limited by the space inside an infected cell, must ensure a sufficient supply of nutrients for replication and survival [4].

To date, it is well established that all studied intracellular apicomplexans and kinetoplastids effectively inhibit apoptotic processes in infected cells. To prevent apoptosis, most pathogens activate host nuclear transcription factor NF-κB, which controls the expression of genes involved in regulating cell death and proliferation [1]. NF-κB, together with inhibitory protein IκB, form an inactive complex within the host cell cytoplasm. Phosphorylation of IκB by a specific IκB-kinase (IKK) causes NF-κB release, 26S proteasome-dependent degradation of IκB, and translocation of the transcription factor into the nucleus followed by activation of specific genes [5]. Apicomplexan parasites exploit different IKKs to activate NF-κB. For instance, *Toxoplasma gondii*, a widespread pathogen of warm-blooded animals and humans, phosphorylates host IκB by means of its own kinase (TgIKK) located at the parasitophorous vacuole membrane. In this case, TgIKK and the kinase of the infected cell are both involved in transcription of NF-kB-activated genes [6]. In contrast to *T. gondii*, piroplasms *Theileria parva* and *Theileria annulata* develop in direct contact with the cytoplasm of infected cells, and they immobilize host IKK on the parasite surface by binding them in a large multisubunit complex [7]. In general, proteins localized on the membranes of the parasite or parasitophorous vacuoles may play an important role in the relationship with the infected cell. As another example, the surface metalloprotease GP63 of the pathogenic trypanosomatid *Leishmania major* inactivates p38, a central component of the host signaling cascade that involves mitogen-activated protein kinases (MAPK) [8].

However, the greatest potential for manipulation with the host machinery should demonstrate the soluble factors that are directly secreted into the infected cytoplasm. Depending on their function, soluble proteins can be specifically trafficked into the host compartment where they can freely interact with the target molecules. Among protozoan intracellular parasites, piroplasms of the genus *Theileria* demonstrate the most interesting examples of such secreted proteins. These parasites directly contact the host cell cytoplasm and cause reversible transformation (immortalization) of infected cells (bovine leukocytes). Five proteins have been experimentally shown to be secreted by *Theileria annulata* into the infected host cell and transported to the nucleus [9], [10], [11], all of which possess a DNA-binding domain and may be involved in controlling host cell growth and differentiation. In addition, a large gene family (85 genes) encoding various secreted subtelomeric proteins has been detected in the *Theileria parva* genome [12]. A number of these proteins contain nuclear localization signals, one of which is expressed in mammalian cells and translocates into the nucleus.

For other members of the phylum Apicomplexa, the possibility of releasing protein factors into the host cytoplasm is limited by the parasitophorous vacuole membrane. Therefore, these parasites are forced to use additional mechanisms to overcome this barrier. *T. gondii* phosphatase PP2C-hn [13] and protein kinase ROP16 [14], the rhoptry proteins secreted into the host cell cytoplasm early during the invasion, are eventually localized in the host nucleus [15]. *Leishmania donovani* elongation factor-1α (EF-1α) may serve as an example of proteins released into the host cell cytoplasm by trypanosomatid parasites. After being transported from the phagosome into the infected macrophages by an unknown mechanism, EF-1α activates host Src homology 2 domain-containing tyrosine phosphatase SHP-1 [16], [17].

In contrast to the aforementioned intracellular parasitic protists, proteins secreted into infected cell by microsporidia, another large group of eukaryotic intracellular microorganisms, remains unexplored to date. However, there are many reasons why this group of fungi-related obligate intracellular parasites are an interesting model to study this mechanism. For instance, distribution of microsporidia among most animal taxa suggests a long history of host-parasite relations. In addition, most microsporidia species directly contact infected host cytoplasm. Sequencing of several microsporidial genomes has demonstrated: (1) a strong minimization of parasite cell machinery [18], [19], [20]; (2) the acquisition of unique transporters to exploit host metabolic system [21]; and (3) the presence of predicted signal peptides responsible for the secretion of a number of proteins that might be involved in parasite-host cell relationships [22], [23]. Finally, it is likely that microsporidia, like other intracellular parasites, possess the capacity to inhibit apoptotic pathways in infected host cells by means of unidentified mechanisms [24], [25].

From the *Antonospora locustae* Genome Database (Marine Biological Laboratory at Woods Hole, funded by NSF award number 0135272; http://forest.mbl.edu/cgi-bin/site/antonospora01) and National Center for Biotechnology Information (NCBI), we have chosen to study seven potentially secreted proteins. First, we overexpressed parasite genes in bacteria *E. coli*, followed by raising specific polyclonal antibodies (Abs) against them. Next, immunoblotting, immunofluorescence microscopy, and heterologous expression in the yeast *Pichia pastoris* confirmed secretion of some *A. locustae* proteins into the infected host cell, suggesting their important role in host-parasite relations.

## Results

### Analysis of sequences

We chose seven functionally different *A. locustae* proteins with predicted N-terminal signal peptides (Table 1) for this study. The highest likelihood of secretion was predicted for the *A. locustae* molecular chaperon of the Hsp70 family. The presence of signal peptides in the protein sequence was predicted by all five servers applied in the study (TargetP, SignalP, SIG-Pred, PrediSi, and Signal-3D). Secretion of trehalase and leucine-rich repeat (LRR)-containing proteins of family A was suggested by four programs. The presence of signal peptides in the hexokinase sequence was confirmed by TargetP, SignalP, and Signal-3D, but not by the SIG-Pred and PrediSi servers. The smallest chance to be a member of microsporidia secretome was predicted for α/β-hydrolase, a ricin B-like protein and a member of the second gene family encoding LRR-proteins, family B; their secretion was only forecast by the TargetP program. Analysis of all studied proteins with the TMHMM server demonstrated the absence of transmembrane helices; thus, they can potentially be transported outside of the parasite cell.

**Table 1:** Secretory signal peptide prediction (Y) or absence (N) in studied *Antonospora locustae* proteins.

Protein	ORF^a^	Length (aa)	M.W. (kDa)	Signal peptide predicton servers				
				TargetP	SignalP	SIG-Pred	PrediSi	Signal-3L
Chaperone Hsp70	1066	679	76.0	Y (17)^b^	Y (17)	Y (17)	Y (17)	Y (17)
Trehalase	1770	669	76.6	Y (36)	N	Y (38)	Y (38)	Y (36)
LRR protein (family A)	204	331	36.8	Y (20)	Y (20)	N	Y (20)	Y (20)
Hexokinase	309	471	52.1	Y (18)	Y (12)	N	N	Y (53)
LRR protein (family B)	515	682	78.8	Y (19)	N	N	N	N
α/β-hydrolase	− c	372	41.5	Y (18)	N	N	N	N
Ricin B-like lectin	1551	166	19.2	Y (15)	N	N	N	N

a ORF number presented in *A. locustae* genome DB (*Antonospora locustae* Genome Project; http://forest.mbl.edu/cgi-bin/site/antonospora01, Marine Biological Laboratory at Woods Hole, funded by NSF award number 0135272).

b predicted length of signal peptide is indicated in parentheses.

c ORF was obtained from the NCBI database (accession number AY608637.1).

### Cloning, sequencing, and overexpression of *A. locustae* genes

Polymerase chain reaction (PCR)-amplification of gene copies with specific primers (Table 2) followed by their cloning in pRSET vectors and sequencing has added new information pertaining to *A. locustae* genome data.

**Table 2:** List of primers used for PCR-amplification of full-size genes encoding the studied proteins.

Encoded protein	Gene size (bp)	Restricton sites	Sequence (5′-3′) of primers (forward/reverse)^a^
Chaperone Hsp70	2040	*Bam*HI*/Pst*I	CCATGGATCCATGCTGTTCTGGTTACTAGCTCTC GTCACTGCAGTCAAAGCTCTTCTCTCATTTTCT
Trehalase	2010	*Bam*HI/*Eco*RI	CCATGGATCCATGACACATTTGTTGATCACAAGC GTCAGAATTCTACACCTCAAGGGGAACCAC
LRR protein (family A)	993	*Bam*HI/*Eco*RI	CCATGGATCCATGATCAGAAATGCAGCATGTGT GTCAGAATTCATCTGCCTGATGCTGCTGTGCT
Hexokinase^b^	1416	*Xho*I/*Eco*RI	CCAACTCGAGATGAGGATGCTTTTGATCTTTGC GTCAGAATTCTACTCAACTAAGAAGGAAGC
LRR protein (family B)	2049	*Bam*HI/*Eco*RI	CCATGGATCCATGTATCTGCAGAGAATGATGT GTCAGAATTCTTAGTCACACACGCCATAACTT
α/β-hydrolase	1119	*Bam*HI/*Eco*RI	CCATGGATCCATGCTGCCTGAGATCATCATGAA GTCAGAATTC TTACTCATCAAAAGCAACAACT
Ricin B-like lectin	501	*Bam*HI/*Eco*RI	CCATGGATCCCATGCAAATATATTCTATTCTTA GTCAGAATTCTAATGTAAATTTTTTTTGCGA

a restriction enzyme sites are underlined in the primer sequences.

b to express hexokinase in *Pichia pastoris*, the forward primer with the *Bgl*II site instead of *Xho*I was used for PCR amplification of gene copies.

**Figure 1:**
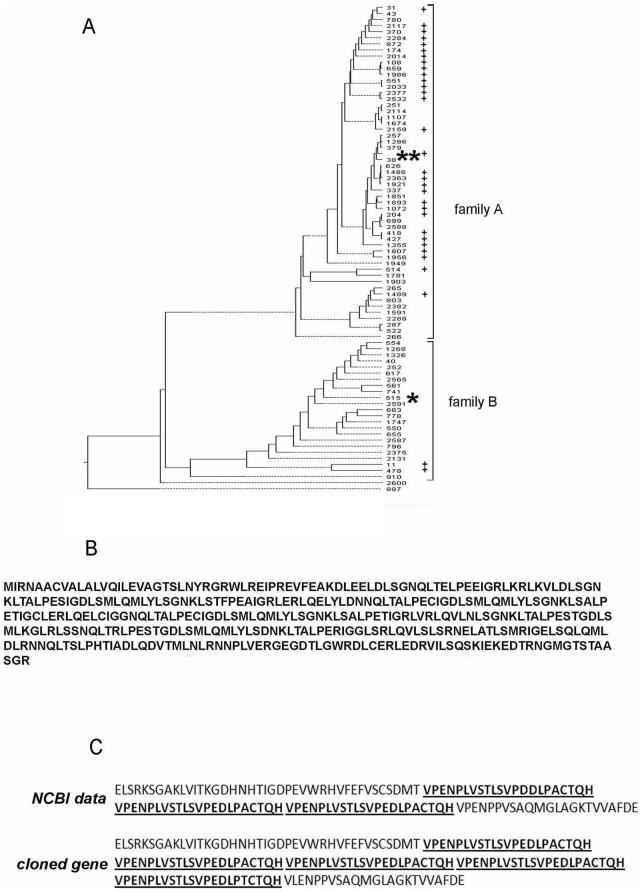
Analysis of ORFs encoding *Antonospora locustae* proteins. A. ClustalW alignment demonstrated that most genes (ORFs) encoding LRR-proteins in *A. locustae* genome form two large families. The location of the new LRR-protein of family A found in the microsporidian genome and expressed in this study is marked with two asterisks. The position of the protein encoded by ORF 515 of family B is indicated by one asterisk. Proteins with secretion signal peptides predicted by SignalP server are indicated by a plus sign. B. The amino acid sequence of the novel *A. locustae* LRR-protein of family A. C. Compared to NCBI data, two extra C-terminal repeats VPENPLVSTLSVP(E/D)DLP(A/T)CTQH were found in cloned α/β-hydrolase.

First, primers designed for amplification of open reading frame (ORF) 204 facilitated the isolation of a new variants of the gene belonging to one of two multigene families encoding LRR-proteins in the parasite genome (Figure 1A). The cloned gene showed 85%–87% identity of amino acid sequence (Figure 1B) to the closest members of multiple family A.

Second, a novel sequence encoding α/β-hydrolase was found in the *A. locustae* genome. The cloned gene possessed five repeats (VPENPLVSTLSVP[E/D]DLP[A/T]CTQH) in the C-terminal portion of the protein, and the sequence available from the NCBI (AY608637.1) demonstrates three copies of the same repetitive peptide (Figure 1C). The predicted molecular weight of α/β-hydrolase encoded by this new gene was approximately 46.2 kDa, whereas the sequence from GeneBank only encodes a 41.5 kDa protein.

The sequences of other cloned genes or gene fragments were identical to ORFs presented in the *A. locustae* genome project. Sodium dodecyl sulfate polyacrylamide gel electrophoresis (SDS-PAGE) analysis has shown that molecular weights of recombinant products expressed in *E. coli* correspond to the predicted sizes plus a 4 kDa vector tag. The exception is the discrepancy between the predicted (50 kDa) and estimated SDS-PAGE (66 kDa) size of recombinant α/β-hydrolase (not shown). Among the studied *A. locustae* proteins, α/β-hydrolase has the lowest isoelectric point (4.75), the most negative charge at pH 7 (−25.08), and almost 2-times higher acidic amino acid content (60 residues) compared to basic ones (33 residues). Thus, slow migration of the enzyme may be explained by the fact that acidic proteins bind less SDS [26].

### Western blot assay of *A. locustae* proteins in infected host cells

**Figure 2:**
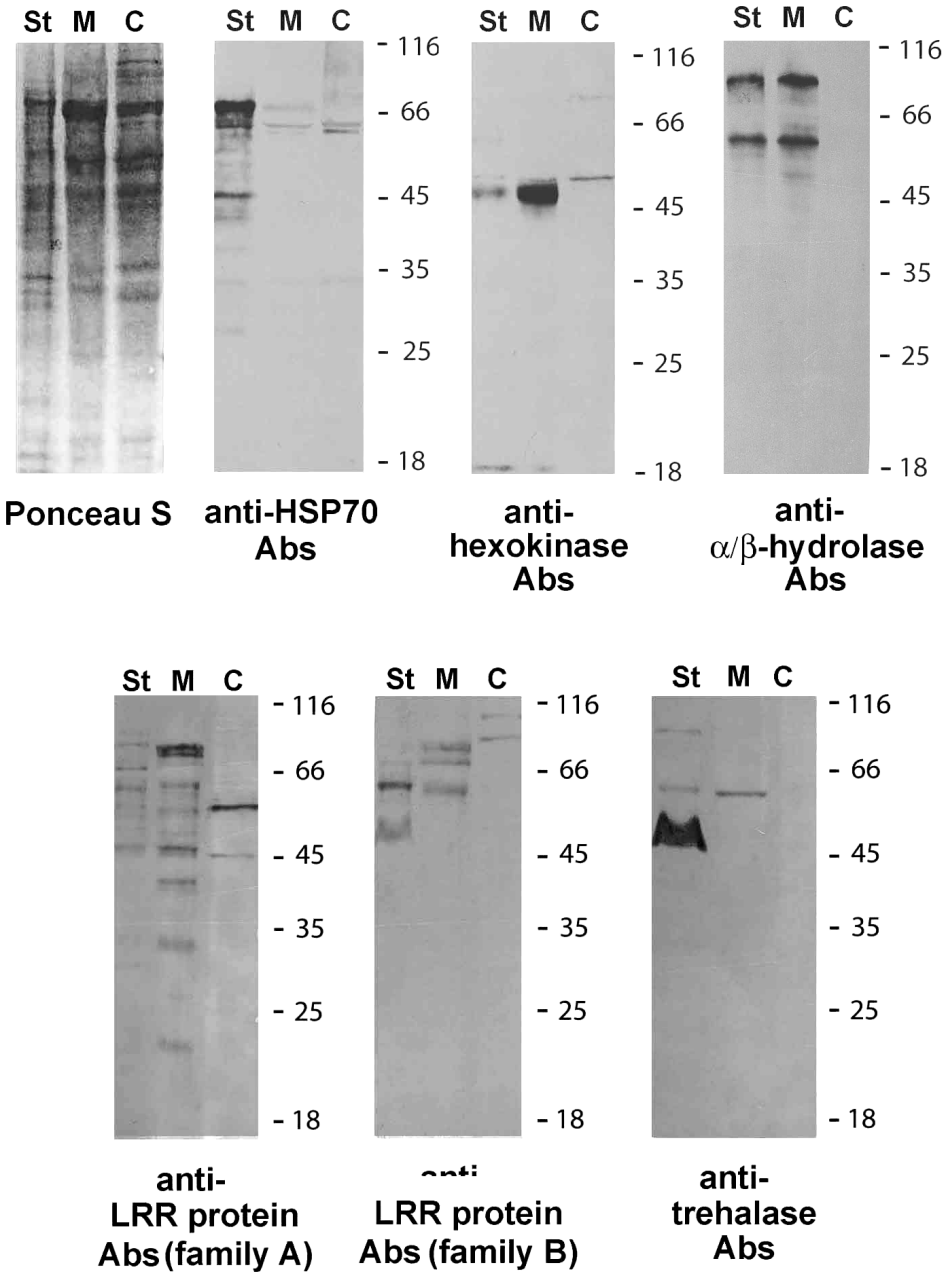
Western blot assay of *A. locustae* proteins. Samples of *A. locustae* intracellular stages isolated in Percoll density gradient (lane St), infected host cytoplasm obtained by gentle homogenization of locust fat bodies following sedimentation of parasites (lane M), and cytoplasm of uninfected (control) fat bodies prepared in the same manner (lane C) were equalized in protein concentration. The proteins transferred on nitrocellulose membranes were stained either by Ponceau S or by Abs against *A. locustae* Hsp70, hexokinase, α/β-hydrolase, two LRR-proteins, and trehalase. This experiment identified parasite proteins in the infected host cytoplasm.

To determine whether the studied proteins are secreted into the infected host cell, we raised Abs against recombinant products and prepared the following samples for immunoblotting equalized in protein concentration: proteins of *A. locustae* intracellular developmental stages isolated by centrifugation in a Percoll density gradient (Figure 2, lane St), proteins of infected host cell cytoplasm obtained by gentle homogenization of locust fat bodies following sedimentation of parasites (Figure 2, lane M), and samples of cytoplasm of uninfected (control) fat bodies prepared in the same manner (Figure 2, lane C). Western blot analysis showed specific accumulation of some microsporidian proteins in the infected host cytoplasm.

#### Ricin B-like lectin

Abs raised against ricin-like lectin intensely stained a major 19 kDa protein in the soluble fraction of *A. locustae* spore homogenate (not shown). Neither the samples from intracellular stages nor those from the infected or control host cytoplasm were decorated with anti-ricin B Abs.

#### Molecular chaperone Hsp70

Despite the reliable prediction of the signal peptide, the molecular chaperone Hsp70 did not accumulate in the host cell cytoplasm. However, the protein was found in the parasite cells isolated by Percoll centrifugation. Like the most homologous chaperons of other microsporidia species, *A. locustae* Hsp70 possesses a C-terminal sequence REEL which retains proteins in the endoplasmic reticulum (ER) lumen [27]. Because the other three Hsp70 chaperones found in *A. locustae* genome lack any secretion signal and C-terminal specific sequence, there is a good reason to assume that this form resides in the ER lumen providing the correct folding of translocated polypeptides.

#### Hexokinase

Western blot analysis of the prepared samples with hexokinase-specific Abs clearly demonstrated accumulation of a 48 kDa protein in the cytoplasm of infected cells, its absence in control fat bodies, and low content in the intracellular stages of the parasite. As evaluated by SDS-PAGE, the molecular weight of the identified band was similar to the size of *E. coli*-expressed recombinant hexokinase (about 52 kDa) minus the N-terminal vector tag (not shown). Unfortunately, we cannot accurately estimate the extent of the cleavable signal peptide because different programs predict various values (Table 1).

#### α/β-hydrolase

Immunoblotting with Abs against recombinant α/β-hydrolase showed accumulation of the protein in the samples of infected host cells and *A. locustae* intracellular stages. Abs recognized two bands of 62 kDa and over 80 kDa in both samples. The lower band corresponded to the size of the recombinant protein (65–66 kDa) minus the N-terminal vector tag (4 kDa). The band of high molecular weight may conform to a form with additional copies of C-terminal VPENPLVSTLSVP(E/D)DLP(A/T)CTQH repeats found in the enzyme molecule.

#### LRR-proteins (family A)

The multiplicity of sequences encoding LRR-proteins in the *A. locustae* genome and high identity between members of two families suggests that specific Abs should detect multiple bands in the sample of infected host cells. Moreover, the similarity of microsporidia LRR-proteins with regulatory proteins (GTPases, GTP-binding proteins, adenylate cyclases, transcriptional regulators, protein kinases, and scribbled homologs) suggests a low concentration in the samples. As expected, Abs produced against LRR-proteins similar to the one encoded by ORF 204 (family A) recognized several faint bands of about 20–105 kDa in infected host cells. This result is consistent with the genomic data from family A proteins predicting sizes ranging from 17.2 kDa (ORF 1956) to 104.4 kDa (ORF 872; *A. locustae* Genome Database).

#### LRR-proteins (family B)

Abs to the LRR-protein encoded by ORF 515 (family B) showed specific accumulation of three proteins from 63 kDa to 80 kDa in the adipocyte cytoplasm of infected locusts. Because the protein encoded by ORF 515 has a predicted size of 78.8 kDa, and its nearest neighbors are of similar sizes (63.3 kDa [ORF 617], 67.5 kDa [ORF 2265], 68.1 kDa [ORF 778], 84.6 kDa [ORF 741], and 85.8 kDa [ORF 561]), there is good reason to believe that the three detected bands belong to LRR-proteins of family B.

#### Trehalase

As the predicted size of *A. locustae* trehalase is 76.6 kDa, and four programs predicted the presence of a 36–38 residue long signal peptide (Table 1), and thus the molecular weight of the mature enzyme should be around 72 kDa. Immunoblotting with Abs against recombinant trehalase did not reveal the presence of a detectable band of such a size in the samples of prespore developmental stages, mature spores, or infected and uninfected host cells. However, the anti-trehalase Abs reacted to a 64 kDa protein in infected cell cytoplasm. A similar protein was also present in the stages of intracellular development, though at much lower concentrations.

### Immunolocalization of *A. locustae* hexokinase and α/β-hydrolase in host cells

**Figure 3:**
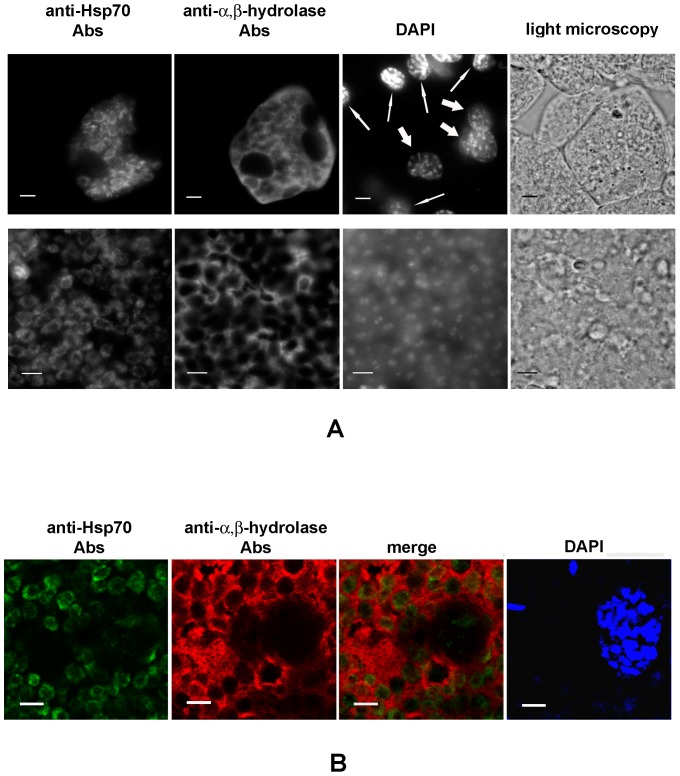
Immunolocalization of *A. locustae* α/β-hydrolase in infected cells. A. Accumulation of microsporidial enzymes in the infected cytoplasm was demonstrated using wide field immunofluoresence microscopy. The top row shows the infected zone with three host nuclei surrounded by uninfected cells. The bottom row shows a portion of an infected cell at higher magnification. The thick and thin arrows on the DAPI image indicate nuclei of infected and uninfected host cells, respectively. Scale bars, 10 μm (top row) and 5 μm (low row). B. Colocalization of *A. locustae* α/β-hydrolase and Hsp70 was analyzed by confocal microscopy. Scale bars, 5 μm.

**Figure 4:**
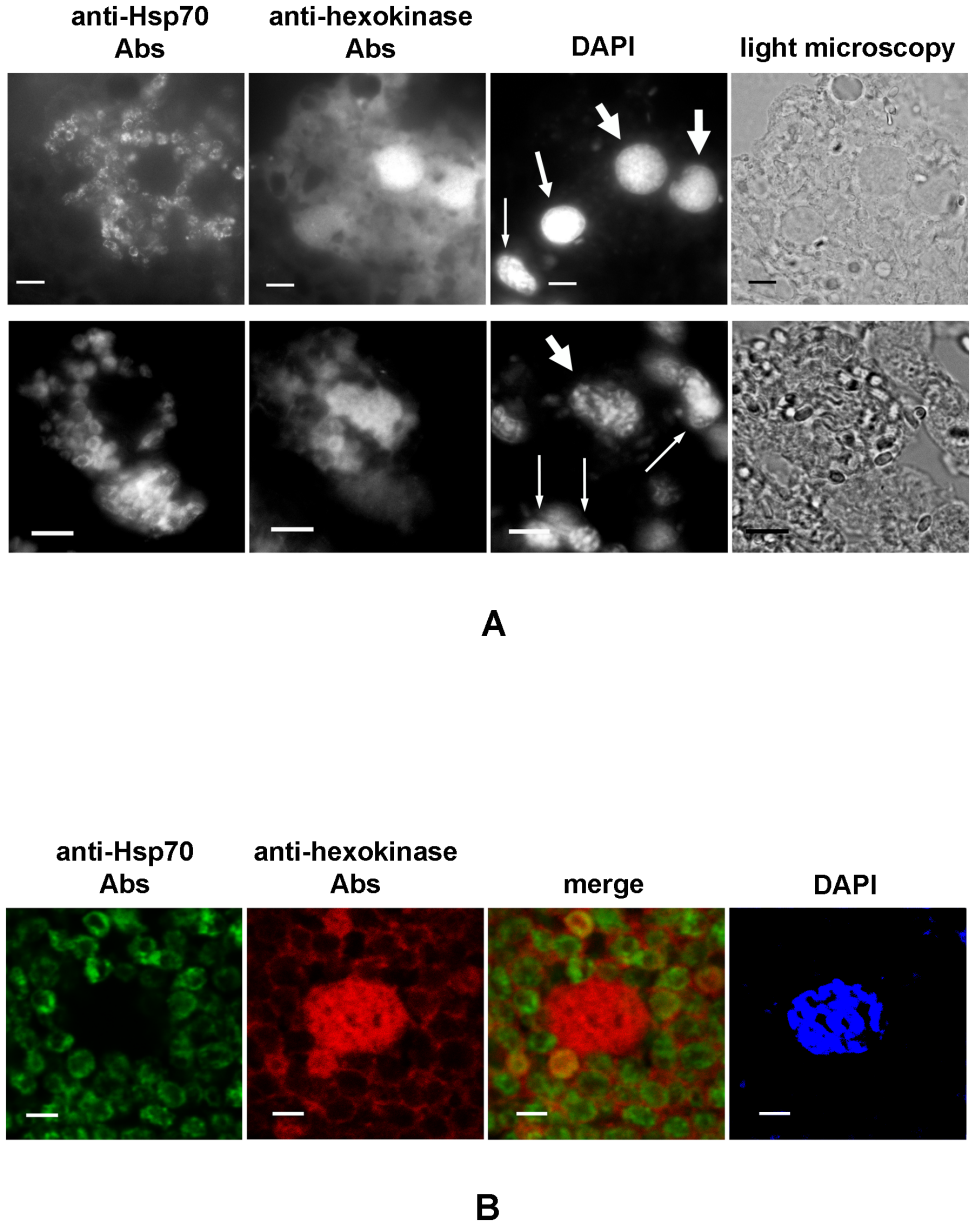
Immunolocalization of *A. locustae* hexokinase in infected cells. A. The presence of microsporidial protein in infected cytoplasm and its accumulation in host nuclei was demonstrated using wide field immunofluoresence microscopy. The top row demonstrates accumulation of hexokinase in three nuclei of infected host cells. A nucleus of an uninfected cell is located in the bottom left corner. The bottom row shows accumulation of hexokinase in the single nucleus of an infected cell surrounded by a number of uninfected ones. The thick and thin arrows on the DAPI images indicate nuclei of infected and uninfected host cells, respectively. The nucleus of infected cells on the periphery of the invasion region (top row) is marked by the middle arrow. Scale bars, 10 μm. B. Colocalization of *A. locustae* hexokinase and Hsp70 were analyzed by confocal microscopy. Scale bars, 5 μm.

To localize the major proteins recognized by Abs against hexokinase and α/β- hydrolase, we performed immunofluorescence analysis (IFA) of 10 μm frozen sections of fat bodies of infected locusts. Abs raised against the expressed *A. locustae* chaperone Hsp70 were used to visualize microsporidia cells and to localize non-secreted parasite proteins. As expected, IFA confirmed accumulation of *A. locustae* Hsp70 inside parasite cells. In the intracellular developmental stages, anti-Hsp70 Abs patchily stained membranous structures around the nucleus (Figures 3, 4). Anti-Hsp70 Abs did not recognize any structures in the cytoplasm of infected or uninfected (control) host cells. Impermeable to the Abs, spores also remained unstained, which confirms the intrasporal localization of the chaperone.

In contrast, anti-α/β-hydrolase Abs did not recognize microsporidia on sections of infected tissue. Meronts, sporonts, sporoblasts, and spores, like host cell nuclei, were visualized as dark zones of unstained material surrounded by bright halos of stained host cytoplasm. Uninfected fat body cells were not labeled by the anti-α/β-hydrolase Abs (Figure 3).

Abs against hexokinase also reacted with the cytoplasm of infected host cells. In these sections, the intracellular stages of parasites were also stained, and the staining was occasionally even a little brighter than the surrounding host cytoplasm. As expected, mature spores and uninfected host cells were not stained. The most interesting outcome of our study was the intense decoration of the nuclei of infected cells with the anti-hexokinase Abs (Figure 4). The brightest labeling was observed over the nuclei in heavily infected cells in the center of the invasion zone. The fluorescence of the nuclei decreased at the periphery of the infected region in less infected cells (Figure 4A, top row, nucleus marked by the middle arrow), and was absent in the nuclei of uninfected cells.

Attempts to obtain a specific signal with Abs against LRR-proteins and trehalase in infected cells were unsuccessful. This may be due to low content of proteins secreted by parasite, and a variety of bands recognized by these Abs in host and parasite cells (Figure 2).

### *Pichia pastoris* cells recognize and secrete *A. locustae* proteins

**Figure 5:**
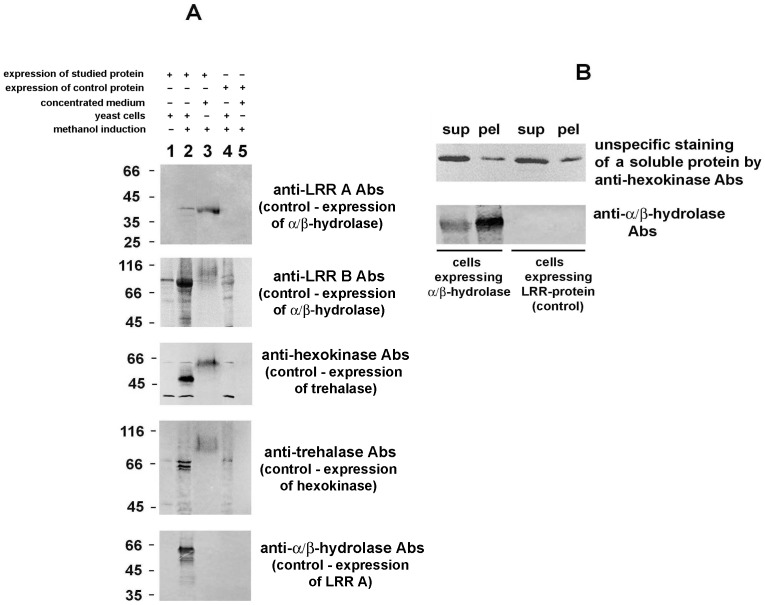
Heterologous expression of *A. locustae* proteins in yeast *P. pastoris* cells. A. Expression of microsporidian proteins without any exogenous signal peptides in yeast *P. pastoris* led to the secretion of two LRR-proteins, hexokinase and trehalase. Lane 1, yeast cells before methanol induction; lane 2, cells expressing the studied protein after adding methanol; lane 3, culture medium concentrated approximately 50 times; lane 4, yeast cells expressing another parasite protein after methanol induction (control cells); lane 5, concentrated medium after cultivation of cells expressing another parasite protein (control medium). Samples were analyzed by immunoblotting with Abs specific to the expressed protein. B. Though α/β-hydrolase was not found in the culture medium, a significant amount of enzyme accumulated in the insoluble fraction of *P. pastoris* cell homogenate. Yeast cells were broken in the presence of 0.3 M sucrose and homogenate was cleared at 270 *g* for 4 minutes and then centrifuged at 18,000 *g* for 20 minutes. Western blot analysis of the supernatant (lane “sup”) and pellet (lane “pel”) showed precipitation of the enzyme, which suggests its association with cell membranes.

With the phylogenetic fungi-microsporidia relationship in mind, we expressed full-size copies of genes encoding *A. locustae* α/β-hydrolase, hexokinase, trehalase, and both LRR-proteins in the methylotrophic yeast *P. pastoris* (strain GS115). To determine whether *P. pastoris* cells recognize secretion signals in the parasite proteins, we chose the vector pPIC3.5 which lacks any signal peptide and was designed specifically for intracellular expression of foreign genes. Western blot analysis of yeast cells and concentrated culture media showed methanol-induced expression of parasite genes in *P. pastoris* as well as secretion of all expressed proteins, except for α/β-hydrolase (Figure 5A).

The most efficient secretion was observed for the LRR-protein of family A. Concordance of the protein size assessed by SDS-PAGE with the predicted value (42 kDa) suggests an absence or very low content of carbohydrates in the secreted molecules. In the case of expression of another LRR-protein (family B), Abs recognized the protein of the predicted size (about 79 kDa) in *P. pastoris* cell extracts. In the concentrated medium, Abs detected diffuse bands of a larger size that may correspond to the glycolsylated form of this protein.

Expression *of A. locustae* hexokinase and trehalase was manifested by the appearance of bands of approximately 48 kDa and 64 kDa in yeast cells, respectively. Interestingly, the mobility of both bands coincided with the mobility of proteins detected in the infected host cytoplasm. In the concentrated culture media, anti-hexokinase and anti-trehalase Abs stained the larger bands, which may be due to the glycosylation of secreted molecules.

The absence of α/β-hydrolase in the yeast culture medium was an unexpected result of the expression experiment, as it contradicts the IFA data. Interestingly, the sizes of the protein accumulated in yeasts was similar to the size of the lower band of the enzyme revealed in microsporidia-infected fat bodies (Figure 5A). Destruction of yeast cells in the presence of 0.3 M sucrose followed by centrifugation of cleared homogenate and Western blot analysis of the supernatant and pellet showed precipitation of the enzyme with the membrane fraction (Figure 5B). Thus, it is possible that the protein entered into the yeast secretory pathway. Since α/β-hydrolase is the most intensely expressed parasite protein accumulating in *P. pastoris* cells, the assemblage of unfolded molecules within the ER lumen could be a possible obstacles preventing its secretion.

## Discussion

In this study, we demonstrated that microsporidia *A. locustae* are able to secrete a variety of functionally distinct proteins into infected host cells. First, we detected parasite hexokinase in the host cells. The same mobility of the recombinant form expressed in *E. coli*, methanol-induced band in methylotrophic yeast *P. pastoris*, and the protein detected in infected host cells confirmed that we were, in fact, identifying parasite hexokinase.

Previously, we demonstrated that hexokinase activity was absent from isolated spores and prespore stages of a microsporidian *Paranosema grylli*, while the activities of other glycolytic enzymes were detectable [28], [29]. Recently, it was shown that hexokinase of the microsporidian *Nematocida parisii* is highly expressed at early stages of parasite intracellular development, unlike other glycolytic enzymes [22]. In addition, a *S. cerevisiae* secretion trap system was used to demonstrate the ability of secretion signals of six microsporidian hexokinases to direct secretion in a fungal system [22]. These data suggest that the parasite enzyme may be secreted out of the microsporidian cell, and could perform an additional function, aside from participation in glycolysis.

Herein, we have demonstrated secretion of a full-size *A. locustae* hexokinase by *P. pastoris* and revealed the accumulation of the parasite's enzyme in the nuclei of infected host cells. Interestingly, the fragment MWKWVSDIIKL of *A. locustae* hexokinase, flanked by 164 and 176 residues, matches the consensus ΦX_2-3_ΦX_2-3_ΦXΦ (where Φ· is Leu, Ile, Val, Phe, or Met, and X indicates any amino acid residue) representing the nuclear export sequence (NES) recognized by the nuclear export receptor CRM1 (Xpo1) [30]. This result is in a good agreement with the data illustrating that *S. cerevisiae* hexokinase II is translocated to the nucleus in the presence of excess glucose and participates in the regulation of glucose metabolism [31]. Nuclear localization of hexokinase HXKII was demonstrated in HeLa cells by immunocytochemistry and subcellular fractionation [32]. Thus, localization of microsporidian hexokinase in host nuclei suggests an additional function of the enzyme associated with the regulation of transcription of host genes involved in carbohydrate metabolism. Alongside regulation of gene transcription, hexokinase can directly phosphorylate hexoses, which mobilizes host carbohydrates and facilitates the rapid growth of parasites. It has been reported that microsporidia cause depletion of host glycogen and rapid uptake of glucose by the infected cell [33].

Another interesting enzyme that accumulated in the infected host cells was a member of the α/β-hydrolase family, which includes such hydrolytic enzymes as proteases, lipases, peroxidases, esterases, epoxide hydrolases, etc [34]. The presence of C-terminal proline-rich repeats in the *A. locustae* enzyme suggests its functional relatedness to mammalian bile salt-stimulated lipases (BSSL), which are multifunctional lipolytic enzymes that exhibit a number of carboxyl ester hydrolase activities [35]. The finding of two bands of *A. locustae* hydrolase in the samples of parasite and infected host cells resembles the heterogeneity in molecular weight from 100 to 160 kDa described for human milk-derived BSSL. These variations are due to the presence of deletions or insertions in a hypervariable region of the gene encoding C-terminal proline-rich repeats [36]. A depletion of fat reserves during microsporidia infection is well known [37], [38]. Therefore, secretion of parasite lipase into host cells can be expected.

Among the minor proteins detected in the cytoplasm of infected host cells, we identified a 64 kDa band recognized by Abs against *A. locustae* trehalase. Though the apparent molecular weight of the band differed from what was predicted, it was similar to the weight of heterologous trehalase that accumulated in yeast. Further identification of this protein seems to be promising because trehalose is the major sugar of insect hemolymph. It is synthesized in the fat body and released into hemolymph with the help of specific trehalose transporters [39]. Hence, secretion of parasite trehalase into infected cytoplasm should redirect the flow of carbohydrates and increase the rate of glucose metabolism in the host cell.

It should be noted that proteomic analysis has previously detected trehalase in microsporidia *Trachipleistophora hominis* and *Spraguea lophii* spores [40], [23], while in the current study we failed to find this enzyme in *A. locustae* spores by immunoblot. This might be explained by varying levels of the enzyme in spores of different microsporidia species. Previously, Undeen and Vander Meer [41] demonstrated that spore germination in aquatic microsporidia is accompanied by the loss of trehalose and an increase in glucose concentration. Meanwhile, no changes in sugar content were observed during germination of spores of terrestrial species. Interestingly, in contrast to the three enzymes involved in trehalose biosynthesis, trehalase was not detected in the proteome of *Encephalitozoon cuniculi* spores [42].

Alongside metabolic enzymes, regulatory proteins appear to be secreted by microsporidia, as we detected some LRR-proteins in *A. locustae* infected host cells. In addition, both microsporidian LRR-proteins expressed in *P. pastoris* were secreted by yeast cells. To date, large families of LRR-proteins were found in several distantly related microsporidian species, and many of them demonstrate the presence of secretion signal peptides and the absence of transmembrane domains [23]. There are two arguments in favor of the assumption that miscrosporidian LRR-proteins may interfere with the regulatory cascades of the host cell. First, as LRR-proteins are often involved in protein-protein interactions, they may form inactive complexes with host regulatory molecules disturbing functional dimer-monomer cycles [23]. Second, many of microsporidian LRR-proteins demonstrate similarity to GTPases, GTP-binding proteins, adenylate cyclases, transcriptional regulators, protein kinases, etc. Further functional analysis of LRR-proteins secreted by microsporidia should involve identification of their targets in the host cell.

## Methods

### Ethics Statement

This study was carried out in strict accordance with the recommendations in the Guide for the Care and Use of Laboratory Animals of the National Institutes of Health, Guidelines for polyclonal antibody production (Animal Care and Use Committee, University of California, Berkeley) and Institutional Guidelines (December 23, 2010). The protocol was approved by the Bioethics Committee of I.M. Sechenov Institute of Evolutionary Physiology and Biochemistry Russian Academy of Sciences (Protocol of the Bioethics Committee session 4 of 17.01.2013 approved by the order of the director of IEPhB RAS N 375-k-a of 12.10.2010).

### Insects and parasites

Maintaining a laboratory culture of migratory locust *Locusta migratoria migratorioides* R. & F, *per os* infection of third-instar nymphs with *A. locustae* spore suspension and preparation of insect fat body were done according to procedures described by Sokolova and Lange [43]. *A. locustae* stages of intracellular development and mature spores were isolated from the fat bodies of the artificially infected locusts by centrifugation in a Percoll density gradient as described previously [44].

### Sequence analyses

The presence of secretion signal peptides in the studied proteins was assessed with the help of following servers: SignalP 4.0 (http://www.cbs.dtu.dk/services/SignalP/), TargetP (http://www.cbs.dtu.dk/services/TargetP/), SIG-Pred (http://www.bioinformatics.leeds.ac.uk/prot_analysis/Signal.html), PrediSi (http://www.predisi.de/index.html) and Signal-3D (http://www.csbio.sjtu.edu.cn/bioinf/Signal-3L/). The transmembrane topology of proteins was evaluated by TMHMM server (http://www.cbs.dtu.dk/services/TMHMM/). Alignment of *A. locustae* LRR-proteins was performed in MegAlign (DNASTAR Lasergene) using ClustalW.

### DNA constructs for expression of *A. locustae* proteins

The sequences encoding studied proteins were obtained from the *A. locustae* Genome Database (Marine Biological Laboratory at Woods Hole, funded by NSF award number 0135272; http://forest.mbl.edu/cgi-bin/site/antonospora01). The sequence of the *A. locustae* α/β-hydrolase gene was obtained from the NCBI. The specific primers for PCR amplification of the full-size gene copies contained the restriction enzyme sites (Table 2) for insertion of DNA fragments into pRSET and pPIC3.5 vectors (Life Technologies, Carlsbad, CA, USA) designed for expression of foreign proteins in bacteria *E. coli* and yeast *P. pastoris*.

For bacterial expression, all studied genes were amplified by PCR with *Pfu*-polymerase using the parasite genomic DNA template as previously described [45]. The products were gel purified, cleaved with the appropriate restriction enzymes, and inserted into a pRSET vector that was digested using the same enzymes. The PCR-amplified Hsp70 gene was digested at the *Bam*HI site in forward primer and at the *Eco*RI inner site (position 1063). A 1068 bp fragment of the parasite chaperone was cloned into the pRSETA plasmid. The *A. locustae* trehalase gene was cut using *Bam*HI/*Kpn*I restriction enzymes and a 682 bp 5′-fragment was inserted into the pRSETA plasmid. The ORF515 sequence encoding one of the LRR-proteins was digested at the *Pst*I (position 401) and the *Hind*III (position 1546) sites and a 1155 bp fragment was cloned into the pRSETC vector. Full-size copies of genes encoding α/β-hydrolase and the second LRR-protein were cloned into the pRSETA plasmid at the *BamH*I/*EcoR*I sites. The hexokinase gene was inserted into the same vector at the *Xho*I/*Eco*RI sites. The sequence encoding ricin-like lectin was cloned at the *Bam*HI/*Eco*RI sites into the pRSETB vector. T7 forward and reverse primers were used for sequencing of cloned DNA fragments.

To express α/β-hydrolase, trehalase, and the two LRR-proteins in *P. pastoris*, full-length genes were inserted into plasmid pPIC3.5 at the *Bam*HI/EcoRI sites. In the case of hexokinase expression, a gene copy amplified with *Bgl*II-containing the forward primer was cloned into a pPIC3.5 plasmid digested with *Bam*HI/*Eco*RI. The overhang sequences resulting from *Bam*HI and *Bgl*II digest are complementary.

### Heterologous expression in *E. coli* and isolation of recombinant proteins

Expression was induced in the BL21(DE3)-derived C41 *E. coli* strain [46] as previously described [45]. After cultivation at 37°C, bacterial cells were harvested by centrifugation at 3,000 *g* for 10 minutes and sonicated in 50 mM Tris-Cl buffer (pH 8.0). Recombinant proteins, except α/β-hydrolase, were accumulated as insoluble inclusion bodies (IBs) in bacteria. After sedimentation at 1,500 *g* for 10 minutes and careful washing, the IBs formed by Hsp70, both LRR-proteins, and ricin B-like lectin were easily solubilized in a solution of 50 mM Tris-Cl (pH 8.0) and 8 M urea. IBs formed by recombinant trehalase and hexokinase were effectively dissolved in the presence of 2% SDS and 1% 2-ME because urea solution was incapable of solubilizing them. The solubilization procedure was followed by the removal of insoluble debris at 18,000 *g* for 10 minutes. Soluble α/β-hydrolase that accumulated in the bacterial cytoplasm was purified by immobilized metal ion affinity chromatography (IMAC) as it was previously described for the mitochondrial form of *A. locustae* Hsp70 [47].

### Production and purification of polyclonal Abs

Immunization of rabbits and mice with isolated proteins thoroughly dialyzed against TBS (50 mM Tris-HCl [pH 7.4], 150 mM NaCl) and raising immune sera and purification of polyclonal Abs on nitrocellulose membrane strips with transferred recombinant antigen were performed as previously described [45], [47].

### Heterologous expression of *A. locustae* proteins in *P. pastoris*

For transformation of *P. pastoris* cells (strain GS115), constructs were linearized using *Sac*I or *Sal*I restriction enzymes. DNA was purified using the standard phenol/chloroform procedure, and this was followed by ethanol precipitation. Electroporation was performed using the Electroporator 2510 (Eppendorf, Germany). After electroporation, cells were immediately resuspended in 1 M sorbitol, precipitated by centrifugation, and plated on MD agar medium containing 1.34% YNB, 4×10^−5^% biotin, 2% dextrose, and incubated for 2 days at 28°C. To check the recombination of linearized constructs into *P. pastoris* genome, different colonies were spheroplasted by zymolyase [48] and used as a template for PCR with primers for corresponding genes. Positive colonies were transferred to BMGY medium (1% yeast extract, 2% peptone, 100 mM potassium phosphate buffer (pH 6.0), 1,34% YNB, 4×10^−5^% biotin, 1% glycerol) and cultivated for 2 days at 28°C. Cultivated cells were precipitated at 1,200 *g* for 10 minutes, transferred to medium BMMY (1% yeast extract, 2% peptone, 100 mM potassium phosphate buffer [pH 6.0], 1,34% YNB, 4×10^−5^% biotin, 0.5% methanol) and cultivated for another 2 days at 28°C.

The pelleted yeast cells and medium concentrated about 50 times with Centricon centrifugal filter units (EMD Millipore, Bellerica, MA, USA) were analyzed by SDS-PAGE and immunoblotting. Heating of yeast cell pellets suspended in 50 mM Tris-HCl buffer (pH 8.0) at 95°C for 10 minutes with an equal volume of 2× SDS-PAGE sample buffer was sufficient for release of *P. pastoris* inner proteins.

To verify α/β-hydrolase association with yeast membranes, cells after methanol-induced expression were broken by vortexing with 2.5 mm glass balls (BDH, United Kingdom) in TS solution (50 mM Tris-Cl [pH 8.0] and 0.3 M sucrose) for 30 minutes. Homogenate was cleared at 270 *g* for 4 minutes and then centrifuged at 18,000 *g* for 20 minutes. The supernatant was transferred to another tube, while the pellet was washed in TS and resuspended with the volume of TS that brought the volume up to that of the final supernatant and both samples were assayed by immunoblotting.

### Preparation of samples of host and *A. locustae* proteins for immunoblotting

Fat bodies of experimentally infected and control *Locusta migratoria* locusts were gently disrupted in phosphate-buffered saline (PBS; 138 mM NaCl, 3 mM KCl, 1.5 mM KH_2_PO_4_, 8 mM Na_2_HPO_4_, pH 6.8) using a glass homogenizer and a freely matched Teflon pestle. Spores and intracellular stages of microsporidia were pelleted at 100 *g* for 10 minutes, the supernatant was further centrifuged at 18,000 *g* for 20 minutes, and the soluble fraction was analyzed by immunoblotting. Purified parasite cells were ruptured by sonication. Spores were broken in TS solution by vortexing with 2.5 mm glass balls for 30 minutes and the resulting homogenate was cleared by centrifugation at 100 *g* for 10 minutes. Protein concentration in the samples was determined by Bradford's method [49].

### SDS-PAGE and immunoblotting

The protein samples were heated at 95°C for 10 minutes with an equal volume of 2× sample buffer (125 mM Tris-HCl, pH 6.8, 4% SDS, 10% 2-mercaptoethanol, 20% glycerol) and separated by SDS-PAGE in 12% gel [50]. For immunoblotting, the separated proteins were transferred to nitrocellulose membranes according to the instructions for the electroblotting apparatus (Mini-PROTEAN, Bio-Rad Laboratories, Hercules, CA, USA). The membranes were blocked for 1 hour in TTBS (TBS with 0.05% Tween-20) with 1% BSA, washed with TTBS, and incubated overnight at 4°C with purified Abs diluted 1∶1000. After washing with TTBS, the membranes were incubated for 1 hour with goat anti-rabbit Abs conjugated with peroxidase (Bio-Rad Laboratories) diluted 1∶4000 in TTBS, consequently washed with TTBS and TBS, and the peroxidase reaction was performed with 4-chloro-1-naphthol (Sigma-Aldrich, St Louis, MO, USA) as a substrate.

### Immunofluorescence microscopy

Fat bodies of infected locusts were fixed in PBS with 4% formaldehyde, washed in PBS containing 50 mM glycine, incubated in 30% sucrose for cryoprotection, and frozen in liquid nitrogen. Frozen sections (10 μm thickness) were prepared with the Microm HM 520 cryotome and placed on microscope slides. The sections were blocked in TTBS with 1% bovine serum albumin and incubated overnight at 4°C with Abs diluted 1∶50 in blocking solution. For co-localization of parasite proteins, Abs against *A. locustae* Hsp70 were raised in mice. Anti-α/β-hydrolase and anti-hexokinase Abs were raised in rabbits. After washing with TTBS, slides were incubated for 2 hours at room temperature with Alexa Fluor 488 Anti-Mouse IgG and Alexa Fluor 546 Anti-Rabbit IgG Abs (Life Technologies), diluted 1∶50 in TTBS, and washed with the same solution. Cell nuclei were stained with 4′,6-diamidino-2-phenylindole (DAPI). The preparations in Vectashield mounting medium (Vector Laboratories, Burlingame, CA, USA) were observed with the help of a Carl Zeiss AxioImager M1 fluorescent microscope and a Leica TCS SP5 MP confocal microscope.
